# Salivary Melatonin and MMP-9 Levels in Stage III Periodontitis: A Pilot Study Using Standardized Pre-Sleep Saliva Collection

**DOI:** 10.3390/medicina62091762

**Published:** 2026-09-13

**Authors:** Ivan Ivanov, Emilia Naseva, Antoaneta Mlachkova, Velitchka Dosseva-Panova, Zdravka Pashova-Tasseva, Hristina Maynalovska, Boyan Kirilov, Viktoria Petrova, Nikolay Ishkitiev, Sonia Apostolova

**Affiliations:** 1 Department of Periodontology, Faculty of Dental Medicine, Medical University of Sofia, 1431 Sofia, Bulgaria; a.mlachkova@fdm.mu-sofia.bg (A.M.); v.doseva@fdm.mu-sofia.bg (V.D.-P.); z.pashova@fdm.mu-sofia.bg (Z.P.-T.); h.maynalovska@fdm.mu-sofia.bg (H.M.); 2 Department of Health Management and Health Economics, Faculty of Public Health “Prof. Tzecomir Vodenitcharov, MD, DSc”, Medical University of Sofia, 1431 Sofia, Bulgaria; e.naseva@foz.mu-sofia.bg; 3 Institute of Experimental Morphology, Pathology and Anthropology with Museum, Bulgarian Academy of Sciences, Acad. G. Bonchev Str., Bl. 21, 1113 Sofia, Bulgaria; drkirilov@yandex.com; 4 Department of Conservative Dentistry, Faculty of Dental Medicine, Medical University of Sofia, 1431 Sofia, Bulgaria; victoria.petrova@fdm.mu-sofia.bg; 5 Department of Chemistry and Biochemistry, Medical Faculty, Medical University of Sofia, 1431 Sofia, Bulgaria; nishkitiev@medfac.mu-sofia.bg; 6 Institute of Biophysics and Biomedical Engineering, Bulgarian Academy of Sciences, Acad. G. Bonchev Str., Bl. 21, 1113 Sofia, Bulgaria; sonia_apostolova@yahoo.com

**Keywords:** stage III periodontitis, salivary biomarkers, melatonin, matrix metalloproteinase-9, unstimulated saliva, inflammation, ROC analysis

## Abstract

*Background and Objectives*: Periodontitis is a chronic multifactorial inflammatory disease characterized by progressive destruction of the tooth-supporting tissues. Although periodontal diagnosis is primarily based on clinical and radiographic parameters, salivary biomarkers may provide additional biological information on inflammatory activity and host-response regulation. Matrix metalloproteinase-9 (MMP-9) is involved in extracellular matrix degradation and periodontal tissue destruction, whereas melatonin is a circadian-related molecule with antioxidant, anti-inflammatory, and immunomodulatory properties. However, evidence regarding the simultaneous assessment of salivary MMP-9 and melatonin using standardized pre-sleep saliva collection remains limited. This pilot study aimed to evaluate salivary melatonin and MMP-9 concentrations in periodontal health and stage III periodontitis, to assess their interrelationship, and to explore their preliminary in-sample ability to distinguish periodontal health from stage III periodontitis. *Materials and Methods*: This cross-sectional pilot study included 18 systemically healthy non-smoking adults: 9 periodontally healthy participants and 9 patients with stage III periodontitis. Pre-sleep unstimulated saliva was collected between 23:00 and 24:00 h or immediately before sleep, following a standardized protocol. Salivary MMP-9 and melatonin concentrations were determined using enzyme-linked immunosorbent assay. Periodontal diagnosis was established according to the 2017 World Workshop classification. Group comparisons, correlation analyses, and receiver operating characteristic curve analyses were performed as exploratory analyses. *Results*: Salivary melatonin concentrations were significantly lower in patients with stage III periodontitis than in periodontally healthy participants (*p* = 0.019), with a large effect size (Hedges’ g = 1.261). Salivary MMP-9 concentrations were higher in the periodontitis group, but the difference was not statistically significant (*p* = 0.465). No significant correlation was found between salivary melatonin and MMP-9 levels. Receiver operating characteristic analysis suggested preliminary in-sample discriminatory ability for melatonin (AUC = 0.827; *p* = 0.019), whereas MMP-9 showed limited and non-significant discriminatory ability (AUC = 0.593; *p* = 0.508). *Conclusions*: In this pilot sample, lower pre-sleep salivary melatonin levels were associated with stage III periodontitis, whereas MMP-9 showed limited standalone discriminatory ability. These findings should be interpreted as preliminary and hypothesis-generating. The present study does not establish a clinically applicable diagnostic cut-off or validated diagnostic utility for salivary melatonin. Larger, independently validated studies are needed to confirm these observations.

## 1. Introduction

Periodontitis is a chronic multifactorial inflammatory disease characterized by progressive destruction of the tooth-supporting tissues. The 2017 World Workshop classification introduced staging and grading as a framework for defining disease severity, complexity of management and risk of progression. According to this classification, stage III periodontitis represents an advanced form of periodontal breakdown, associated with substantial attachment loss, deep periodontal lesions, potential tooth loss, and increased treatment complexity [1]. Beyond local periodontal destruction, periodontitis is increasingly discussed as a chronic inflammatory condition with potential systemic relevance, although the present study did not assess systemic inflammatory or vascular outcomes.

Although periodontal diagnosis is still primarily based on clinical and radiographic parameters, increasing attention has been directed toward salivary biomarkers that may provide additional biological information on inflammatory activity and tissue destruction [2]. In this context, saliva has emerged as a particularly attractive diagnostic fluid because it is non-invasive, easily obtainable, and contains locally and systemically derived molecules relevant to periodontal inflammation. Previous reviews have emphasized the potential clinical value of salivary biomarkers in periodontal disease, while systematic evidence suggests that selected molecular markers in saliva may help distinguish periodontitis from periodontal health [3,4].

More recent systematic evidence has further supported the relevance of salivary biomarkers in periodontal research, while also emphasizing the methodological heterogeneity of available studies and the need for standardized sampling, assay validation, and independent confirmation before clinical implementation. In particular, recent systematic reviews have highlighted MMP-9 among salivary inflammatory and tissue-destruction-related markers investigated in periodontitis, although its standalone diagnostic performance may vary across study designs, populations, and analytical protocols [5]. In parallel, salivary proteomic studies and systematic reviews suggest that multi-marker approaches and targeted validation are likely to be necessary for clinically meaningful periodontal biomarker assessment [6].

Matrix metalloproteinases are central proteolytic effectors and key enzymatic drivers of extracellular matrix degradation and periodontal tissue breakdown. In periodontitis, increased MMP expression and activity, together with dysregulation of endogenous tissue inhibitors of metalloproteinases, contribute to excessive degradation of periodontal extracellular matrix components and irreversible loss of periodontal supporting tissues [7]. Among these enzymes, matrix metalloproteinase-9 (MMP-9) is a host-derived gelatinase involved in extracellular matrix degradation and has been implicated in periodontal connective tissue breakdown [8]. In salivary diagnostics, MMP-9 has been identified among salivary biomarkers with potential diagnostic value for periodontitis; however, according to the meta-analysis by Arias-Bujanda et al. [3], MMP-9 demonstrated the lowest sensitivity but the highest specificity among the analyzed salivary markers. Therefore, its standalone diagnostic value should be interpreted cautiously and may vary according to study design, population characteristics, disease definition, and sampling protocols.

MMP-9 was selected for the present pilot study as an exploratory salivary marker related to extracellular matrix degradation and tissue-destructive inflammatory activity. This selection was not intended to imply superiority over MMP-8, which is also highly relevant to periodontal tissue destruction and has been extensively investigated in periodontal diagnostics. Rather, MMP-9 was chosen because previous periodontal research has directly evaluated salivary MMP-9 together with salivary melatonin, which aligned with the specific aim of simultaneously assessing these two biologically distinct molecules in standardized pre-sleep saliva samples. Importantly, MMP-9 was not used in the present study to define the stage of periodontitis. Stage III periodontitis was determined using established clinical and radiographic criteria, whereas MMP-9 was investigated as an exploratory salivary marker related to tissue-destructive and inflammatory biological activity.

Melatonin is a biologically distinct salivary marker. It is primarily known as a central mediator of circadian regulation, and its evening rise is commonly used to assess circadian phase. Dim light melatonin onset is regarded as a reliable marker of the central circadian clock when assessed in plasma or saliva [9]. Beyond its chronobiological role, melatonin also exhibits antioxidant, anti-inflammatory and immunomodulatory properties, which may be relevant in chronic inflammatory conditions [10], including periodontal disease.

In the periodontal context, available evidence suggests that melatonin may be associated with periodontal health and disease, potentially reflecting the balance between oxidative stress, inflammation and host protective mechanisms. Clinical studies have reported altered salivary and gingival crevicular fluid melatonin levels across different periodontal conditions [11], and a systematic review has summarized the potential role of melatonin in periodontal disease through antioxidant and immunomodulatory mechanisms [12].

Recent evidence has directly linked salivary melatonin and MMP-9 in periodontal disease. Bayırlı et al. [13] reported significantly higher salivary MMP-9 levels and significantly lower salivary melatonin levels in periodontitis compared with gingivitis and periodontal health. They also observed positive correlations between clinical periodontal parameters and salivary MMP-9, negative correlations between clinical periodontal parameters and salivary melatonin, and a negative correlation between salivary MMP-9 and melatonin levels.

However, several aspects remain insufficiently clarified. First, despite increasing evidence on salivary biomarkers in periodontitis, the simultaneous evaluation of salivary MMP-9 and melatonin has been scarcely investigated, particularly in well-defined stage III periodontitis. Second, because melatonin secretion is strongly time-dependent and sensitive to light exposure, standardized pre-sleep saliva collection is particularly relevant, but remains underrepresented in periodontal biomarker studies. Third, it is still unclear whether these two biologically different markers—one related primarily to inflammatory tissue destruction and the other to circadian-associated antioxidant and anti-inflammatory regulation—show a direct relationship when assessed under standardized pre-sleep sampling conditions. Finally, the preliminary in-sample ability of salivary melatonin and MMP-9 to distinguish periodontal health from stage III periodontitis remains insufficiently explored and requires cautious investigation in pilot studies before validation in larger cohorts.

Therefore, the present pilot study was designed to compare salivary MMP-9 and melatonin levels between periodontally healthy individuals and patients with clinically diagnosed stage III periodontitis using standardized pre-sleep unstimulated saliva collection. The study aimed to evaluate between-group differences in both biomarkers, to determine whether a statistically significant relationship exists between salivary MMP-9 and melatonin, and to explore their preliminary in-sample ability to distinguish periodontal health from stage III periodontitis.

Based on previous evidence, we hypothesized that patients with stage III periodontitis would exhibit higher salivary MMP-9 levels and lower pre-sleep salivary melatonin levels than periodontally healthy individuals. We further hypothesized that salivary MMP-9 and melatonin would show an inverse association [12,13].

## 2. Materials and Methods

### 2.1. Study Design and Population

This pilot cross-sectional study included adult participants with either periodontal health or stage III periodontitis. The initial sample consisted of 27 participants, including 13 periodontally healthy individuals and 14 patients with periodontitis. Because this was a pilot feasibility study, the sample size was determined by the availability of eligible participants and laboratory resources rather than by a formal a priori power calculation.

All participants were recruited and examined according to predefined eligibility criteria. The study was designed to compare salivary matrix metalloproteinase-9 (MMP-9) and melatonin levels between periodontal health and stage III periodontitis using standardized pre-sleep unstimulated saliva collection.

The study protocol was approved by the Ethics Committee of the Medical University of Sofia (Protocol No. 08/24 April 2025). The study was conducted in accordance with the ethical principles of the Declaration of Helsinki. All participants provided written informed consent prior to inclusion. The study was not prospectively registered.

### 2.2. Eligibility Criteria

Participants were eligible for inclusion if they were systemically healthy non-smokers, did not regularly use systemic medications, had not received periodontal therapy within the previous 6 months, and had not taken systemic antibiotics within the previous 6 months.

These criteria were applied in order to reduce the potential influence of systemic, medication-related, behavioral, and treatment-related factors on the investigated salivary biomarkers.

### 2.3. Final Analytical Sample

The initial laboratory dataset included 27 saliva samples: 13 from periodontally healthy participants and 14 from participants with periodontitis. One participant was classified as stage IV periodontitis and was not included in the present stage III-focused analysis. Among the remaining participants, 8 samples did not yield a numerical salivary melatonin concentration suitable for quantitative analysis because of non-quantifiable or analytically unreliable melatonin results.

Among these eight excluded samples, four belonged to the stage III periodontitis group and showed below-range melatonin results. Four belonged to the periodontal-health group: two showed below-range melatonin results, while two were excluded because of marked variability between the three internal replicate measurements, with some replicate readings falling below the quantification range. Thus, the excluded melatonin values reflected low or analytically unstable low-range measurements rather than values above the calibration range.

Therefore, the final paired biomarker analysis was performed on 18 participants with available numerical values for both salivary melatonin and MMP-9: 9 periodontally healthy participants and 9 patients with stage III periodontitis. No otherwise valid observations were excluded solely to obtain equal group sizes.

**Figure 1:**
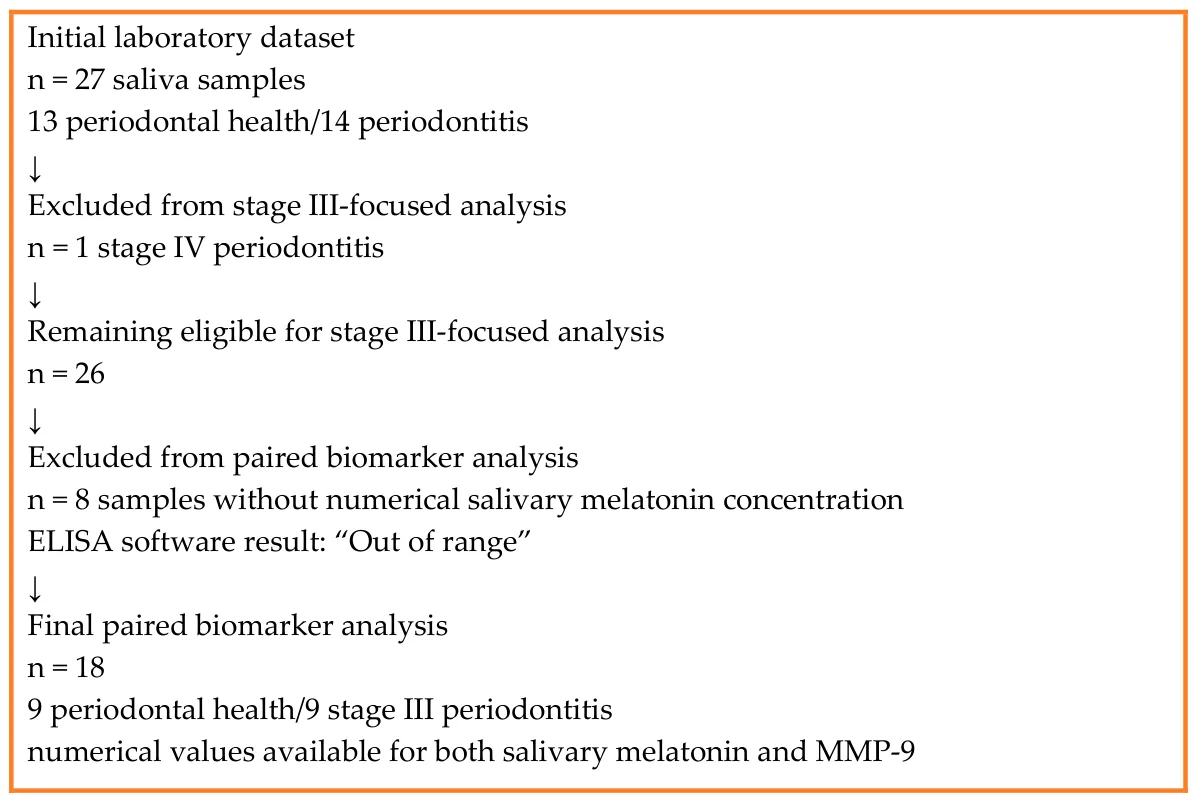
Participant-flow diagram showing the derivation of the final paired biomarker analytical sample. The initial laboratory dataset included 27 saliva samples. One participant with stage IV periodontitis was excluded from the stage III-focused analysis, and eight samples were excluded from the paired biomarker analysis because the ELISA calculation software did not provide numerical salivary melatonin concentrations and reported the results as “Out of range”. The final paired biomarker analysis included 18 participants with available numerical values for both salivary melatonin and MMP-9. Arrows indicate the sequential flow from the initial laboratory dataset to the final paired biomarker analytical sample.

MMP-9 measurements were technically available for all initially analyzed samples. Therefore, an exploratory sensitivity analysis was additionally performed for MMP-9 using all technically valid MMP-9 data from stage III periodontitis patients and periodontally healthy controls. The final analytical sample is presented in Figure 1.

### 2.4. Saliva Collection Protocol

Unstimulated whole saliva was used for laboratory analysis. Saliva samples were collected by the participants at home according to written standardized instructions. Samples were collected approximately 1 h before habitual bedtime. For most participants, this corresponded to the interval between 23:00 and 24:00 h; however, in participants with an earlier bedtime, samples could be collected earlier. Therefore, the protocol should be interpreted as standardized pre-sleep saliva sampling within a nighttime collection window, rather than as a formal dim-light melatonin onset or circadian-phase assessment. The exact clock time of collection was not recorded for each participant, and collection was not performed under controlled lighting conditions.

Participants received written instructions to avoid food and fluid intake before sample collection and to refrain from using screen-emitting electronic devices for at least 1 h before providing the sample. They were instructed to brush their teeth for 2 min using only a toothbrush, without toothpaste, mouthwash, or other oral hygiene products. Interdental cleaning devices were not used immediately before sample collection in order to reduce the risk of blood contamination and potentially increased biomarker values. The exact duration of food and fluid avoidance was not objectively recorded for each participant.

Approximately 2–4 mL of unstimulated saliva was collected in a sterile container provided to each participant.

### 2.5. Sample Storage and Laboratory Analysis

After collection, saliva samples were stored overnight in a refrigerator at approximately +4 °C and were delivered the following day for further processing. Because samples were collected in the pre-sleep nighttime interval and delivered the next day, this corresponded to an overnight pre-analytical interval between sample collection and laboratory receipt. After receipt and technical preparation, the samples were stored at −80 °C until laboratory analysis.

Before ELISA analysis, saliva samples were thawed once and centrifuged at 1000× *g* for 20 min at 4 °C. The obtained salivary supernatants were used immediately for laboratory analysis according to the manufacturers’ instructions. Repeated freeze–thaw cycles were avoided.

Salivary concentrations of MMP-9 and melatonin were determined using enzyme-linked immunosorbent assay (ELISA) kits according to the manufacturers’ instructions using salivary supernatants after samples’ centrifugation. MMP-9 concentrations were measured using the Human Matrix Metalloproteinase 9 (MMP9) ELISA Kit (Abbexa Ltd., Cambridge, UK; catalogue No. abx050165), while melatonin concentrations were measured using the Melatonin (MT) ELISA Kit (Abbexa Ltd., Cambridge, UK; catalogue No. abx354613).

Melatonin concentrations were determined using a competitive enzyme-linked immunosorbent assay, in which the signal intensity is inversely proportional to the antigen concentration in the sample. Concentrations were calculated using a five-parameter logistic standard curve. The standard curve ranged from 7.812 to 250 pg/mL and showed good fit (R^2^ = 0.998). A separate analytical limit of detection distinct from the lower standard concentration and a separate lower limit of quantification were not provided in the available assay/run output. Therefore, 7.812 pg/mL was considered the lower boundary of the validated calibration range for this analytical run. Numerical estimates below this lower standard concentration were considered below the validated quantification range, even when the ELISA software provided numerical values. Samples for which the ELISA calculation software did not provide a numerical melatonin concentration and reported the result as “Out of range” were excluded from the final quantitative analysis. Numerical melatonin estimates close to or below the lower standard concentration were retained for exploratory analysis when the software provided a numerical concentration; however, this was acknowledged as an analytical limitation.

All patient samples were analyzed in triplicate. For statistical analysis, numerical concentrations were used when the triplicate measurements provided an interpretable result. Samples were excluded from quantitative melatonin analysis when the software reported the result as “Out of range” or when the internal triplicate measurements showed marked inconsistency that did not allow reliable quantitative interpretation.

For MMP-9 analysis, saliva samples were initially diluted 1:200 before ELISA measurement. Because the manufacturer’s protocol provides recommended dilution guidance for serum or plasma samples but does not specify a dilution ratio for saliva, and because preliminary absorbance readings of the salivary samples exceeded the upper standard range of the assay, an additional dilution/correction factor of 11 was required to bring the calculated concentrations within the interpretable analytical range. Therefore, final MMP-9 concentrations were back-calculated using a total correction factor of 2200 and expressed as ng/mL. According to the manufacturer’s specifications, the analytical sensitivity of the MMP-9 assay was 0.1 ng/mL, the assay range was 0.156–10 ng/mL before correction for sample dilution, and the intra-assay and inter-assay coefficients of variation were <10%. However, intra-assay and inter-assay coefficients of variation were not independently calculated in the present pilot analysis. Patient samples from both periodontal groups were processed according to the same laboratory protocol. Patient samples were analyzed in triplicate. Standards and blanks were run in duplicate. Manufacturer-provided assay parameters were used for interpretation of the ELISA results.

### 2.6. Periodontal Examination and Case Definition

All periodontal examinations were performed by a single experienced examiner at the Department of Periodontology, Faculty of Dental Medicine, Medical University of Sofia, using a standardized clinical protocol. No separate formal intra-examiner calibration or reproducibility assessment was performed for the present pilot study. A full-mouth periodontal examination was conducted with a UNC-15 periodontal probe (Hu-Friedy, Chicago, IL, USA). All erupted teeth, excluding third molars, were examined at six sites per tooth: mesiobuccal, midbuccal, distobuccal, mesiolingual/palatal, midlingual/palatal, and distolingual/palatal. Clinical assessment included probing pocket depth (PPD), clinical attachment level (CAL), and bleeding on probing (BoP).

The clinical examination was supplemented by radiographic assessment of alveolar bone loss to support periodontal diagnosis and staging. Periodontal diagnosis was established according to the criteria of the 2017 World Workshop on the Classification of Periodontal and Peri-Implant Diseases and Conditions [14].

Periodontal health was defined as the absence of clinical periodontitis, with probing pocket depths ≤3 mm and bleeding on probing <10%. Stage III periodontitis was diagnosed according to the 2017 World Workshop classification. The diagnosis was based on interdental clinical attachment loss of ≥5 mm at the site of greatest loss, radiographic bone loss extending to the middle third of the root or beyond, and case-complexity factors such as probing pocket depth ≥6 mm, vertical bone loss ≥3 mm, furcation involvement class II or III, and/or tooth loss attributable to periodontitis, where present and available.

The control group included participants with clinical periodontal health, while the test group included patients diagnosed with stage III periodontitis. This classification ensured a clear distinction between participants without clinical evidence of periodontal inflammatory destruction and patients with advanced periodontal disease.

### 2.7. Statistical Analysis

Statistical analysis was performed according to the type of variables, sample size, and distribution of quantitative data. The normality of distribution was assessed using the Shapiro–Wilk test, considering the small size of the two study groups.

Age was compared between groups using the Mann–Whitney U test. Sex distribution was assessed using Fisher’s exact test. Between-group comparisons of salivary MMP-9 and melatonin levels were performed using independent-samples *t*-tests. Equality of variances was assessed using Levene’s test. When equality of variances was not assumed, Welch’s *t*-test was applied. As exploratory sensitivity analyses, non-parametric Mann–Whitney U tests were additionally performed for the main between-group biomarker comparisons.

Effect size was calculated using Hedges’ g because of the small sample size. Associations between age, salivary melatonin, and MMP-9 were assessed using Spearman’s rank correlation coefficient. Pearson’s correlation coefficient was additionally used as a sensitivity analysis to assess the robustness of the correlation results.

The discriminative ability of the investigated biomarkers was evaluated using receiver operating characteristic (ROC) curve analysis and the area under the curve (AUC). A *p*-value < 0.05 was considered statistically significant. All statistical analyses were performed using IBM SPSS Statistics, version 29. GraphPad Prism version 5.0 (GraphPad Software Inc., La Jolla, CA, USA) was used for graphical visualization. Given the pilot nature of the study and the small final analytical sample, all statistical analyses were considered exploratory. No formal adjustment for multiple testing was performed. Therefore, individual *p*-values were interpreted cautiously and in conjunction with effect sizes, confidence intervals, biological plausibility, and the hypothesis-generating purpose of the study.

## 3. Results

### 3.1. Participant Characteristics

The final analytical sample included 18 participants, comprising 9 periodontally healthy individuals and 9 patients with stage III periodontitis. The two groups did not differ significantly in age. The median age was 48.00 years (IQR: 45.50–54.50) in the periodontal health group and 49.00 years (IQR: 42.00–57.00) in the stage III periodontitis group (Mann–Whitney U = 34.000; Z = −0.578; exact *p* = 0.605).

Sex distribution differed markedly between groups, with a higher proportion of women in the periodontal health group and a higher proportion of men in the stage III periodontitis group (Fisher’s exact test, two-sided *p* = 0.050). Additional sex-based comparisons showed no statistically significant differences in salivary melatonin or MMP-9 levels between men and women. Because of the marked sex imbalance between groups, additional descriptive analyses according to sex were performed. However, given the very small subgroup sizes, especially the presence of only one male participant in the periodontal-health group, these analyses were considered exploratory and insufficient to exclude residual sex-related confounding. Because of the very small final sample size and sparse subgroup distribution, multivariable adjustment was not considered statistically reliable. Participant characteristics are presented in Table 1.

### 3.2. Salivary Melatonin and MMP-9 Levels

Salivary melatonin concentrations were significantly lower in patients with stage III periodontitis than in periodontally healthy individuals. Mean melatonin levels were 22.21 ± 15.22 pg/mL in the periodontal health group and 7.12 ± 5.29 pg/mL in the stage III periodontitis group. Because equality of variances was not assumed, Welch’s *t*-test was applied. The between-group difference was statistically significant (t = 2.809; df = 9.906; *p* = 0.019), with a mean difference of 15.09 pg/mL (95% CI: 3.10–27.08). The effect size was large (Hedges’ g = 1.261).

Among the 18 participants included in the primary paired biomarker analysis, numerical melatonin estimates below the lower standard concentration of 7.812 pg/mL were present in 3 of 9 periodontally healthy participants and 5 of 9 patients with stage III periodontitis. Because these values were below the validated calibration range of the analytical run, an additional exploratory censored sensitivity analysis was performed by setting numerical values below 7.812 pg/mL to 7.812 pg/mL. In this censored analysis, mean melatonin levels remained lower in the stage III periodontitis group than in the periodontal-health group (9.57 ± 3.24 pg/mL vs. 22.99 ± 14.27 pg/mL; Welch’s *t*-test, *p* = 0.023). These findings were consistent with the direction of the primary analysis but should be interpreted cautiously because several values were at or below the lower quantification boundary.

**Figure 2:**
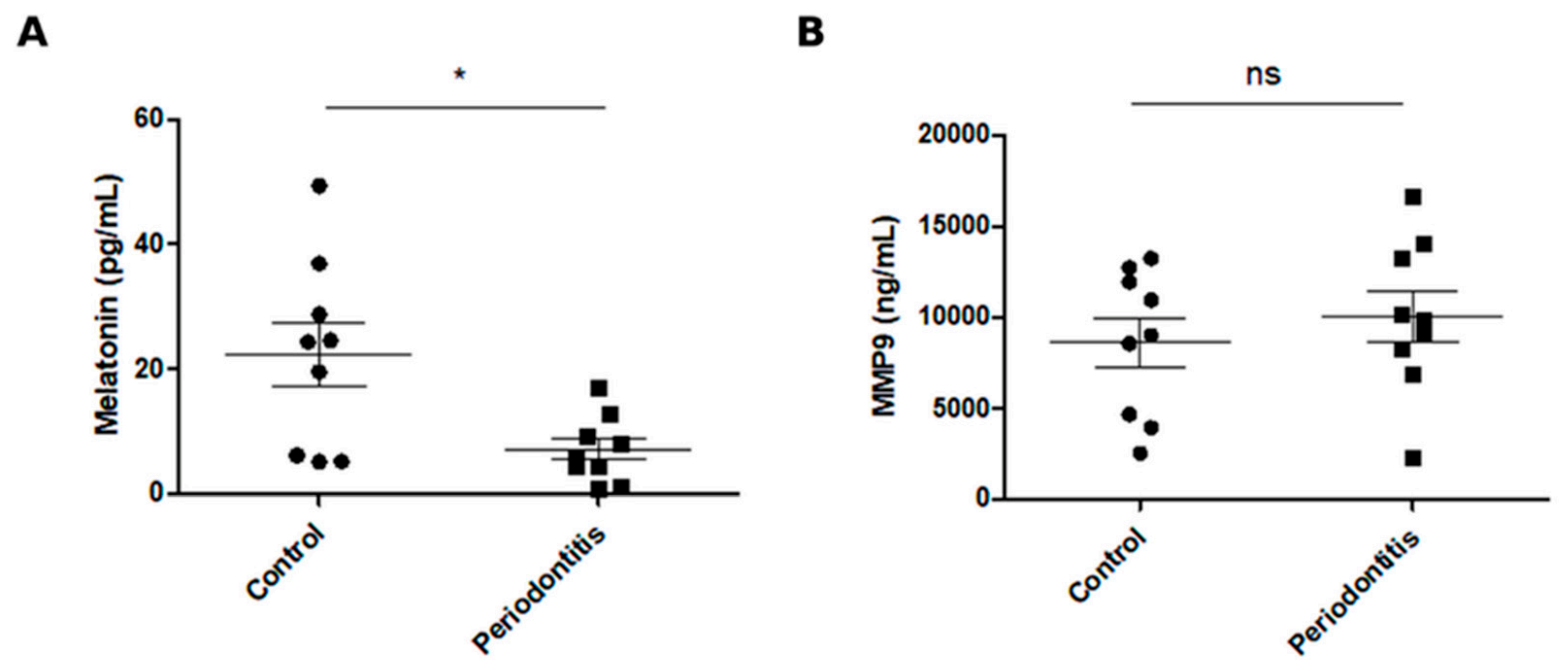
Salivary melatonin and MMP-9 concentrations according to periodontal status. (**A**) Salivary melatonin concentrations were significantly lower in patients with stage III periodontitis than in periodontally healthy participants (*p* = 0.019; Welch’s *t*-test). (**B**) Salivary MMP-9 concentrations were higher in patients with stage III periodontitis, but the difference did not reach statistical significance (*p* = 0.465; independent-samples *t*-test assuming equal variances). Data are presented as individual values with mean ± standard error of the mean (SEM). *: *p* < 0.05; ns, not statistically significant.

Salivary MMP-9 concentrations were higher in patients with stage III periodontitis than in periodontally healthy individuals, but the difference was not statistically significant. Mean MMP-9 levels were 8605.9 ± 4027.7 ng/mL in the periodontal health group and 10,063.3 ± 4238.6 ng/mL in the stage III periodontitis group (t = −0.748; df = 16; *p* = 0.465). The mean difference was −1457.42 ng/mL (95% CI: −5589.16 to 2674.32), and the effect size was small to moderate in magnitude (Hedges’ g = −0.336). Exploratory non-parametric sensitivity analyses were consistent with the primary biomarker findings. The between-group difference in salivary melatonin remained statistically significant using the Mann–Whitney U test (U = 14.0; *p* = 0.022), whereas the between-group difference in MMP-9 remained non-significant (U = 33.0; *p* = 0.536). The individual biomarker distributions are shown in Figure 2, and the corresponding primary between-group comparisons are summarized in Table 2.

In the exploratory sensitivity analysis including all technically valid MMP-9 measurements from periodontally healthy participants and stage III periodontitis patients, mean MMP-9 concentrations were 7289.9 ± 4583.4 ng/mL in the periodontal-health group and 9530.8 ± 4559.8 ng/mL in the stage III periodontitis group. The direction of the difference remained unchanged, with higher MMP-9 values in stage III periodontitis; however, the difference remained statistically non-significant (t = −1.250; df = 24; *p* = 0.223). The mean difference was −2240.9 ng/mL, with a 95% CI of −5941.7 to 1459.9, and the effect size was small to moderate in magnitude (Hedges’ g = −0.475).

### 3.3. Correlation Analysis

Spearman correlation analysis showed no statistically significant association between salivary melatonin and MMP-9 levels (ρ = 0.038; *p* = 0.880). Similarly, Pearson correlation analysis did not demonstrate a significant linear association between the two biomarkers (r = 0.111; *p* = 0.661).

No significant correlations were observed between age and salivary melatonin (ρ = 0.078; *p* = 0.759) or between age and MMP-9 (ρ = 0.105; *p* = 0.679). Thus, within the present pilot sample, the observed biomarker differences were not accompanied by significant age-related associations (Table 3).

### 3.4. ROC Curve Analysis

ROC curve analysis was performed as an exploratory analysis to assess the preliminary in-sample ability of salivary melatonin and MMP-9 to distinguish periodontal health from stage III periodontitis within the present pilot dataset.

For salivary melatonin, lower values were considered indicative of stage III periodontitis. Melatonin showed an AUC of 0.827 (SE = 0.101; 95% CI: 0.629–1.000; *p* = 0.019), suggesting preliminary in-sample separation between the two groups.

An exploratory threshold of ≤18.27 pg/mL was selected as the value maximizing Youden’s index within the present dataset. This threshold corresponded to 100% sensitivity and 66.7% specificity. Accordingly, the sensitivity and specificity values should be regarded as unstable exploratory in-sample estimates. However, because the threshold was derived and evaluated in the same small sample of nine cases and nine controls, and because it was not internally or externally validated, it should be interpreted strictly as a hypothesis-generating estimate and not as a clinically applicable diagnostic cut-off. In contrast, MMP-9 demonstrated limited and non-significant preliminary in-sample discriminatory ability, with an AUC of 0.593 (SE = 0.141; 95% CI: 0.317–0.868; *p* = 0.508) (Table 4).

### 3.5. Overall Summary of Results

Overall, patients with stage III periodontitis demonstrated significantly lower pre-sleep salivary melatonin levels than periodontally healthy individuals, with a large effect size and preliminary in-sample discriminatory ability. MMP-9 showed higher mean values in stage III periodontitis, but without statistical significance and with limited standalone discriminatory ability. No significant correlation was observed between salivary melatonin and MMP-9 levels.

## 4. Discussion

The present pilot study evaluated salivary melatonin and MMP-9 levels in periodontally healthy individuals and patients with stage III periodontitis using standardized pre-sleep unstimulated saliva collection. The main finding was that patients with stage III periodontitis demonstrated significantly lower pre-sleep salivary melatonin concentrations compared with periodontally healthy participants, with a large effect size and preliminary in-sample discriminatory ability. In contrast, salivary MMP-9 levels were higher in the periodontitis group, but the difference did not reach statistical significance. No significant correlation was observed between salivary melatonin and MMP-9 levels. Because the final analytical sample included only nine participants per group, the statistical estimates are imprecise and should be interpreted cautiously. Consequently, the study was underpowered to detect moderate biomarker differences and biomarker-biomarker correlations. In particular, the non-significant difference in MMP-9 and the absence of a significant correlation between melatonin and MMP-9 should not be interpreted as evidence that no biological relationship exists. Rather, these results indicate that such associations could not be reliably demonstrated within the present small exploratory dataset. Accordingly, the statistical analyses should be interpreted as exploratory, with emphasis on effect estimates, confidence intervals, and biological plausibility rather than on binary statistical significance.

The directly observed finding of the present study was an association between stage III periodontitis and lower pre-sleep salivary melatonin levels. This finding should be interpreted in the context of previous literature on the biological properties of melatonin, but it should not be considered direct evidence of altered circadian regulation, impaired sleep, oxidative stress, or a specific anti-inflammatory mechanism. Melatonin is widely recognized as a key mediator of circadian regulation, and its evening rise in saliva is used in chronobiological research for the assessment of circadian phase, particularly through dim-light melatonin onset protocols [9]. Beyond its chronobiological role, melatonin has been associated with antioxidant, anti-inflammatory, and immunomodulatory properties that may be relevant in chronic inflammatory conditions [10]. However, these mechanisms were not directly assessed in the present study.

Previous periodontal studies support a possible association between melatonin and periodontal status. Altered salivary and gingival crevicular fluid melatonin levels have been reported across different periodontal conditions, suggesting that melatonin may be linked to the balance between oxidative stress, inflammation, and host protective mechanisms [11]. A systematic review has also summarized the potential role of melatonin in periodontal disease through antioxidant and immunomodulatory mechanisms [12]. In this context, the significantly lower pre-sleep salivary melatonin levels observed in the present study are consistent with previous evidence suggesting an association between melatonin and periodontal inflammation. However, this finding should not be interpreted as direct evidence of altered circadian regulation, impaired sleep, oxidative stress, or a specific anti-inflammatory mechanism.

It is important to distinguish between findings directly demonstrated by the present data and biological mechanisms proposed to explain them. The present pilot study demonstrated an association between stage III periodontitis and lower pre-sleep salivary melatonin levels, but it did not assess circadian phase, dim-light melatonin onset, sleep quality, oxidative stress, systemic inflammatory mediators, or causal pathways. Therefore, the interpretation that lower melatonin may reflect altered circadian-related antioxidant or anti-inflammatory regulation should be regarded as biologically plausible but hypothesis-generating. Reverse causation and residual confounding by unmeasured behavioral, circadian, psychological, or periodontal factors cannot be excluded. Longitudinal and mechanistic studies are required to determine whether reduced salivary melatonin contributes to periodontal inflammation, results from inflammatory burden, or reflects shared upstream determinants.

Chronic psychological stress may represent one such shared upstream determinant and an important unmeasured confounder. Stress-related neuroendocrine activation may influence nocturnal melatonin secretion, and nighttime physical stress has been shown to blunt the nocturnal melatonin surge in humans. Psychological stress has also been discussed as a potential risk factor associated with periodontitis. Therefore, lower pre-sleep salivary melatonin levels in patients with stage III periodontitis may partly reflect unmeasured stress-related, behavioral, or neuroendocrine factors rather than periodontal inflammation alone [15,16].

Regarding MMP-9, the higher mean values observed in patients with stage III periodontitis are consistent with the biological role of matrix metalloproteinases in periodontal tissue destruction. MMPs are central proteolytic enzymes involved in extracellular matrix degradation and have been implicated in the breakdown of periodontal connective tissues. MMP-9, also known as gelatinase B, participates in the degradation of basement membrane and extracellular matrix components and has been linked to periodontal tissue destruction [7]. Nevertheless, in the present pilot sample, the between-group difference in MMP-9 did not reach statistical significance, and its standalone discriminative performance was limited. The choice of MMP-9 should therefore be interpreted in relation to the exploratory focus of the present study and its comparison with salivary melatonin, rather than as an assumption that MMP-9 is superior to MMP-8 or more suitable for periodontal grading.

This finding does not exclude the biological relevance of MMP-9 in periodontitis. Rather, it suggests that salivary MMP-9 alone may have limited diagnostic informativeness under the conditions of the present pilot study. Previous systematic evidence has identified MMP-9 among salivary biomarkers with potential diagnostic value for periodontitis, but the performance of individual biomarkers may vary according to study design, population characteristics, disease definition and sampling protocol [3]. The lack of statistical significance in the present study may be related to the small sample size, interindividual variability in salivary MMP-9 levels, the single time-point measurement and the strict inclusion criteria, which limited external confounding but may also have reduced biological heterogeneity.

An important finding of the present study was the absence of a significant correlation between salivary melatonin and MMP-9. This differs from the study by Bayırlı et al. [13], who reported significantly higher salivary MMP-9 and lower salivary melatonin levels in periodontitis, as well as a negative correlation between the two biomarkers. The discrepancy may be explained by methodological differences. The referenced study included a broader periodontal spectrum, including periodontal health, gingivitis and periodontitis, whereas the present study compared only periodontal health with a more narrowly defined group of patients with stage III periodontitis. In addition, the small and strictly selected sample may have limited the ability to detect a direct correlation between the two biomarkers.

The correlation analysis should be interpreted with caution because it was based on only 18 observations. The absence of a statistically significant correlation between salivary melatonin and MMP-9 should not be interpreted as evidence that these biomarkers are biologically unrelated. Rather, it indicates that a direct linear or monotonic relationship could not be reliably demonstrated within the present small exploratory dataset. In the context of previous literature, melatonin and MMP-9 may represent different but potentially interconnected biological dimensions of periodontal disease. MMP-9 may be considered primarily related to proteolytic and inflammatory tissue-destructive activity, whereas melatonin may be related to circadian-associated antioxidant and anti-inflammatory regulation. However, these biological interpretations were not directly tested in the present study. Their relationship may therefore be indirect, nonlinear, or mediated by other biological factors, including local inflammatory burden, oxidative stress, cytokine activity, other matrix metalloproteinases, psychological stress, and individual circadian variability. Experimental data suggesting that melatonin may inhibit MMP-9 activity support the biological plausibility of a relationship between these molecules, even if such a relationship is not expressed as a direct correlation in every clinical sample [17]. In addition, unmeasured factors such as usual sleep duration, habitual bedtime, chronotype, shift work, caffeine or alcohol intake, recent changes in sleep schedule, and light exposure before sampling may have influenced pre-sleep salivary melatonin levels.

The ROC analysis provided an additional perspective on the preliminary in-sample discriminatory ability of the two molecules. Pre-sleep salivary melatonin showed preliminary in-sample separation between periodontal health and stage III periodontitis, whereas MMP-9 demonstrated limited and non-significant discriminatory ability. This does not imply that melatonin is a more direct marker of inflammatory tissue destruction than MMP-9. Instead, it indicates that, in the present pilot dataset, lower pre-sleep salivary melatonin was more strongly associated with periodontal status than MMP-9.

The exploratory melatonin threshold of ≤18.27 pg/mL was selected as the value maximizing Youden’s index within the present dataset. However, this threshold was derived and evaluated within the same small dataset, which may lead to optimism and overestimation of discriminatory performance. Therefore, no clinically applicable diagnostic cut-off can be established from the present study. In addition, the comparison involved periodontally healthy individuals and patients with advanced stage III periodontitis, representing a highly separated clinical spectrum. Discriminatory performance in this setting may be higher than would be expected in real-world populations including gingivitis, early-stage periodontitis, or less severe periodontal conditions. In this context, salivary melatonin should be regarded as a preliminary candidate marker associated with periodontal status rather than as a validated diagnostic biomarker. Independent validation in larger, prospectively powered and clinically more diverse cohorts is required before any clinical diagnostic relevance can be considered.

The methodological choice of standardized pre-sleep unstimulated saliva collection was particularly appropriate for the present study. Unstimulated saliva is a non-invasive and clinically applicable biological fluid that may reflect locally and systemically derived biomarkers relevant to periodontal inflammation. At the same time, melatonin secretion is strongly time-dependent and sensitive to light exposure; therefore, standardizing the pre-sleep collection interval and pre-collection conditions was essential to reduce circadian-related variability [18,19]. Nevertheless, the present protocol should not be interpreted as a formal dim light melatonin onset assessment or as an objective sleep evaluation.

In summary, the present pilot study suggests that stage III periodontitis was associated with lower pre-sleep salivary melatonin levels, whereas salivary MMP-9 showed only a non-significant tendency toward higher levels. The absence of a significant correlation between melatonin and MMP-9 indicates that a direct linear or monotonic relationship between these biomarkers could not be demonstrated in this small dataset. These preliminary findings support further investigation of pre-sleep salivary melatonin as a candidate salivary marker associated with periodontal status. Future studies should use larger and demographically balanced samples, repeated saliva sampling, validated sleep and circadian assessment, broader periodontal phenotyping, and independent validation cohorts.

### 4.1. Strengths

The current study has several strengths. First, it focused on a well-defined clinical phenotype by comparing periodontally healthy individuals with patients diagnosed with stage III periodontitis according to the 2017 World Workshop classification, supported by full-mouth periodontal examination and radiographic assessment. Second, the study used a standardized pre-sleep unstimulated saliva collection protocol, which is particularly relevant for melatonin because of its time-dependent secretion. Third, the inclusion of systemically healthy non-smoking participants without recent periodontal therapy or systemic antibiotic use reduced the potential influence of major systemic, behavioral and treatment-related confounders. Finally, the simultaneous assessment of salivary melatonin and MMP-9 allowed evaluation of two biologically distinct molecules relevant to periodontal research: a circadian-related antioxidant/anti-inflammatory molecule and a tissue destruction-related inflammatory marker.

### 4.2. Limitations

Several limitations should also be acknowledged. The main limitation is the pilot design and the small final analytical sample, which included 18 participants. In addition, part of the initially recruited sample was excluded because of analytical limitations in the quantitative determination of salivary melatonin, which reduced the sample size, decreased statistical power, and may have introduced selection bias. No adjustment for multiple testing was performed; therefore, individual *p*-values should be interpreted cautiously and considered exploratory. The correlation analyses were also limited by the small number of observations and should therefore be interpreted as exploratory and underpowered. The absence of statistically significant correlations should not be interpreted as evidence of absence of biological associations. The sex distribution differed markedly between groups, with a predominance of women in the periodontal-health group and men in the stage III periodontitis group. Although exploratory sex-based comparisons did not show statistically significant differences in salivary melatonin or MMP-9 levels, the very small subgroup sizes do not allow residual sex-related confounding to be excluded.

In addition, although periodontal examinations were performed by a single experienced examiner using a standardized full-mouth protocol, no separate formal intra-examiner calibration or reproducibility assessment was performed. Detailed aggregated periodontal inflammatory burden indices, such as mean full-mouth PPD, CAL, BoP percentage, number of sites with deep pockets, and plaque indices, were not available for inclusion in the present analytical dataset. These issues should be considered methodological limitations. Another important limitation is the absence of validated subjective or objective sleep assessment; therefore, the findings should be interpreted as pre-sleep salivary melatonin data and not as direct evidence of sleep disturbance.

Another limitation concerns the saliva collection procedure and the lack of detailed assessment of periodontal, behavioral, and circadian determinants of salivary melatonin. Samples were collected at home according to written instructions and not under direct supervision. Although participants were instructed to collect saliva approximately 1 h before habitual bedtime and to avoid screen-emitting electronic devices before sampling, exact clock time of collection, ambient light exposure, the exact duration of food and fluid avoidance, caffeine or alcohol intake before sampling, and compliance with the pre-collection instructions were not objectively recorded. In addition, usual sleep duration, habitual bedtime, chronotype, shift work, recent changes in sleep schedule, BMI, psychological stress, perceived stress, anxiety, depression, stress-related biomarkers such as cortisol, oral hygiene status, plaque levels, and detailed measures of periodontal inflammatory burden were not systematically assessed or included in the analysis. Therefore, chronic stress cannot be excluded as a potential confounder or shared upstream determinant linking lower pre-sleep salivary melatonin levels and periodontal status. Overall, these variables represent unmeasured potential confounders. The protocol should be interpreted as standardized pre-sleep saliva sampling rather than as a formal dim-light melatonin onset assessment or standardized circadian-phase assessment. These pre-analytical, periodontal, behavioral, and circadian factors may have contributed to variability in salivary melatonin concentrations.

The choice of oral-fluid matrix should also be considered. Saliva was selected because it allows non-invasive, feasible, and repeatable pre-sleep sampling and is suitable for the simultaneous assessment of salivary melatonin and MMP-9 under standardized collection conditions. This was particularly relevant for melatonin, which is commonly assessed in saliva in circadian-related research. However, gingival crevicular fluid may provide more site-specific information on periodontal inflammatory activity, while oral-rinse samples may capture broader oral inflammatory burden and may offer complementary diagnostic information for periodontal biomarkers. Because GCF and oral-rinse samples were not collected in the present pilot study, biomarker performance could not be compared across different oral-fluid matrices. Therefore, the use of saliva alone may have limited the site-specific periodontal diagnostic informativeness of the biomarker measurements.

Another limitation is that MMP-8 was not assessed. Therefore, the present study cannot compare the performance of MMP-9 with MMP-8 or determine whether MMP-8 would have provided stronger discrimination of periodontal status in this sample.

Pre-analytical sample handling should also be considered as a limitation. Saliva samples were stored overnight at approximately +4 °C before further processing and were subsequently stored at −80 °C until laboratory analysis. Although repeated freeze–thaw cycles were avoided and all samples were thawed only once before ELISA analysis, no dedicated stability assessment was performed to determine whether the overnight refrigerated storage period influenced salivary melatonin or MMP-9 concentrations. Therefore, pre-analytical storage conditions may have contributed to biomarker variability and should be more strictly controlled in future studies.

Analytical limitations related to salivary melatonin quantification should also be considered. In addition, some numerical melatonin estimates were close to or below the lower standard concentration of the assay; therefore, these values and the derived ROC threshold should be interpreted cautiously and only as exploratory estimates. The ROC analysis should also be interpreted with caution. The melatonin threshold was derived and evaluated within the same small dataset and was not internally or externally validated. In addition, the comparison included only periodontally healthy individuals and patients with advanced stage III periodontitis. This highly separated clinical spectrum may overestimate discriminatory performance compared with broader clinical populations including gingivitis, early-stage periodontitis, or less severe periodontal conditions. Furthermore, both biomarkers were measured at a single time point, and the cross-sectional design does not allow causal conclusions. Finally, the exploratory melatonin threshold identified in the ROC analysis should be regarded as hypothesis-generating and not as a validated diagnostic cut-off.

## 5. Conclusions

In this exploratory pilot study, lower pre-sleep salivary melatonin levels were associated with stage III periodontitis, while MMP-9 showed only a non-significant tendency toward higher levels. No significant correlation was observed between salivary melatonin and MMP-9.

These findings do not establish causality, altered circadian regulation, impaired sleep, oxidative stress, or validated diagnostic utility of salivary melatonin. The ROC analysis suggested preliminary in-sample separation between periodontal health and stage III periodontitis for melatonin; however, no clinically applicable diagnostic cut-off or validated diagnostic utility can be established from the present dataset. These findings should be interpreted as preliminary and hypothesis-generating. Further studies with larger samples, repeated saliva collection, validated sleep and circadian assessment, and independent validation cohorts are needed to clarify the potential role of pre-sleep salivary melatonin as a candidate marker associated with periodontal status.

## Figures and Tables

**Table 1:** Participant characteristics according to periodontal status.

Variable	Periodontal Health *n* = 9	Stage III Periodontitis *n* = 9	*p*-Value
Age, years, median (IQR)	48.00 (45.50–54.50)	49.00 (42.0–57.00)	0.605
Sex, *n* (%)			0.05
Men, *n* (%)	1 (11.1%)	6 (66.7%)	
Women, *n* (%)	8 (88.9%)	3 (33.3%)	

Data are presented as median (interquartile range, IQR) or number and percentage, *n* (%). Age was compared using the Mann–Whitney U test. Sex distribution was assessed using Fisher’s exact test.

**Table 2:** Between-group comparison of salivary melatonin and MMP-9 levels.

Biomarker	Periodontal Health (*n* = 9), Mean ± SD	Stage III Periodontitis (*n* = 9), Mean ± SD	Mean Difference	95% CI	*p*-Value	Hedges’ g
Melatonin, pg/mL	22.21 ± 15.22	7.12 ± 5.29	15.09	3.10–27.08	0.019	1.261
MMP-9, ng/mL	8605.9 ± 4027.7	10,063.3 ± 4238.6	−1457.4	−5589.2 to 2674.3	0.465	−0.336

Data are presented as mean ± standard deviation (SD). For melatonin, Welch’s *t*-test was used because equality of variances was not assumed. For MMP-9, an independent-samples *t*-test assuming equal variances was applied. Mean differences were calculated as periodontal health minus stage III periodontitis. CI, confidence interval.

**Table 3:** Correlation analysis between age, salivary melatonin, and MMP-9.

Variable Pair	Spearman ρ	*p*
Age and salivary melatonin	0.078	0.759
Age and salivary MMP-9	0.105	0.679
Salivary melatonin and MMP-9	0.038	0.880

ρ, Spearman’s rank correlation coefficient.

**Table 4:** ROC curve analysis of salivary melatonin and MMP-9.

Biomarker	AUC	SE	95% CI	*p*-Value
Melatonin	0.827	0.101	0.629–1.000	0.019
MMP-9	0.593	0.141	0.317–0.868	0.508

AUC, area under the receiver operating characteristic curve; SE, standard error; CI, confidence interval. For melatonin, higher values were associated with periodontal health, whereas lower values were associated with stage III periodontitis. The exploratory threshold was selected by maximizing Youden’s index within the present dataset and should not be interpreted as a validated diagnostic cut-off.

## Data Availability

The data supporting the findings of this study are available from the corresponding author upon reasonable request. The data are not publicly available due to ethical and privacy restrictions.

## Abbreviations

The following abbreviations are used in this manuscript:

AUC	Area under the curve
BoP	Bleeding on probing
CAL	Clinical attachment level
CI	Confidence interval
DLMO	Dim light melatonin onset
ELISA	Enzyme-linked immunosorbent assay
IQR	Interquartile range
MMP-9	Matrix metalloproteinase-9
ROC	Receiver operating characteristic
SD	Standard deviation
SE	Standard error
SEM	Standard error of the mean
